# Understanding immune-mediated cobalt/chromium allergy to orthopaedic implants: a meta-synthetic review

**DOI:** 10.1186/s42836-023-00227-x

**Published:** 2024-02-02

**Authors:** Arnold Chen, Andrew P. Kurmis

**Affiliations:** 1 School of Medicine, University of Adelaide, Adelaide, SA 5000 Australia; 2 Discipline of Medical Specialties, University of Adelaide, Adelaide, SA 5000 Australia; 3 Department of Orthopaedic Surgery, Lyell McEwin Hospital, Elizabeth Vale, SA 5112 Australia; 4 College of Medicine & Public Health, Flinders University, Bedford Park, SA 5042 Australia

**Keywords:** Cobalt, Chromium, Allergy, Hypersensitivity, Orthopaedic implants, Arthroplasty

## Abstract

**Background:**

The frequency of primary joint replacement surgery continues to increase worldwide. While largely considered biologically inert entities, an increasing body of evidence continues to validate a not insignificant incidence of allergic reactions to such implants. Little previous work has explored genuinely immune-mediated reactivity in this context. In the absence of a contemporary published summary on the topic, this paper explored the current state of understanding of cobalt/chromium allergy and proposes a patient management algorithm whereby such immune reactions are clinically suggested.

**Methods:**

A structured, systematic literature review was performed by following PRISMA search principles to provide an updated review of this area.

**Results:**

Thirty-six topic-related articles were identified, the majority reflecting lower tiers of scientific evidence with a lack of homogeneous quantitative data to facilitate valid cohort comparisons. Largely, the available literature represented small case series’ or expert opinions.

**Conclusions:**

Despite increasing clinical awareness and acknowledgement of true allergy to joint replacement components, this review highlighted that the evidence base underpinning the diagnosis and management of such patients is limited. Both patient-reported metal allergy or skin patch testing are grossly unreliable methods and show almost no correlation with true immune reactivity. Recent studies suggested a potential role for patient-specific in vitro cellular activation testing and/or targeted genetic testing when cobalt/chromium allergy is clinically suspected. However, while likely representing the contemporary “best available” approaches both can be costly undertakings, are not yet universally available, and still require broader validation in non-research settings before wider uptake can be championed.

## Introduction

Cobalt and chromium are commonly used metals in the production of a wide range of conventional joint replacement prostheses, including those of the hip, knee, shoulder, ankle and elbow [1]. Most of such implants are composites of a number of blended metals (i.e., alloys) whereby the specific ratios of each are tailored in an attempt to provide a balance between component strength, durability, wear characteristics, stiffness and low reactivity under biological conditions [2]. As none of these metals are naturally occurring substances within the human body, the potential for allergic response by the host (i.e., patients) to any individual substrate is theoretically possible. While certain metals have been suggested to be less immunogenic than others, both cobalt and chromium (either separately or when used in combination) are acknowledged to be responsible for immune-mediated adverse reactions in a recognized subset of patients. With millions of individual metal-based joint replacement components implanted globally each year [3], even small incidences of such reactions may translate to huge patient morbidity. The recent publication by Desai and colleagues (2019) [4] reported that the community prevalence of cobalt and chromium was in the order of 6.43% and 11.58%, respectively. The consequences of true allergy to in situ joint replacements included pain, swelling, decreased functional performance and patient dissatisfaction.

Adverse local tissue reaction (ALTR) is a broad non-specific term used to describe complications secondary to adverse reactions between an implanted object and surrounding biological materials. The further sub-definition of adverse reaction to metal debris (ARMD) is a clinical description in which exposure to ionic and/or particulate materials from metal implants specifically results in a local tissue reaction. Although often used interchangeably (inappropriately) with both ALTR and ARMD, aseptic lymphocyte-dominated vasculitis-associated lesions (ALVALs) are a specific pathology, seen histologically in periprosthetic tissue specimens, resulting from a chronic hypersensitization to the materials of the prosthesis. Recent literature has suggested that ALVALs may be indicative of immune-mediated reaction to metals within the prostheses, rather than simply an irritative reaction [2, 5, 6].

In the absence of a readily available current summation, this paper aimed to review the current literature regarding in situ allergy to cobalt and/or chromium metals when used as substrates within modern joint replacements and to summarize the evidence underpinning such diagnosis. Based on the findings, this work also aimed to propose an investigation and management pathway to guide clinicians treating individual patients in whom a genuine allergy to either the cobalt and/or chromium is considered likely.

## Search strategy and selection criteria

**Fig. 1:**
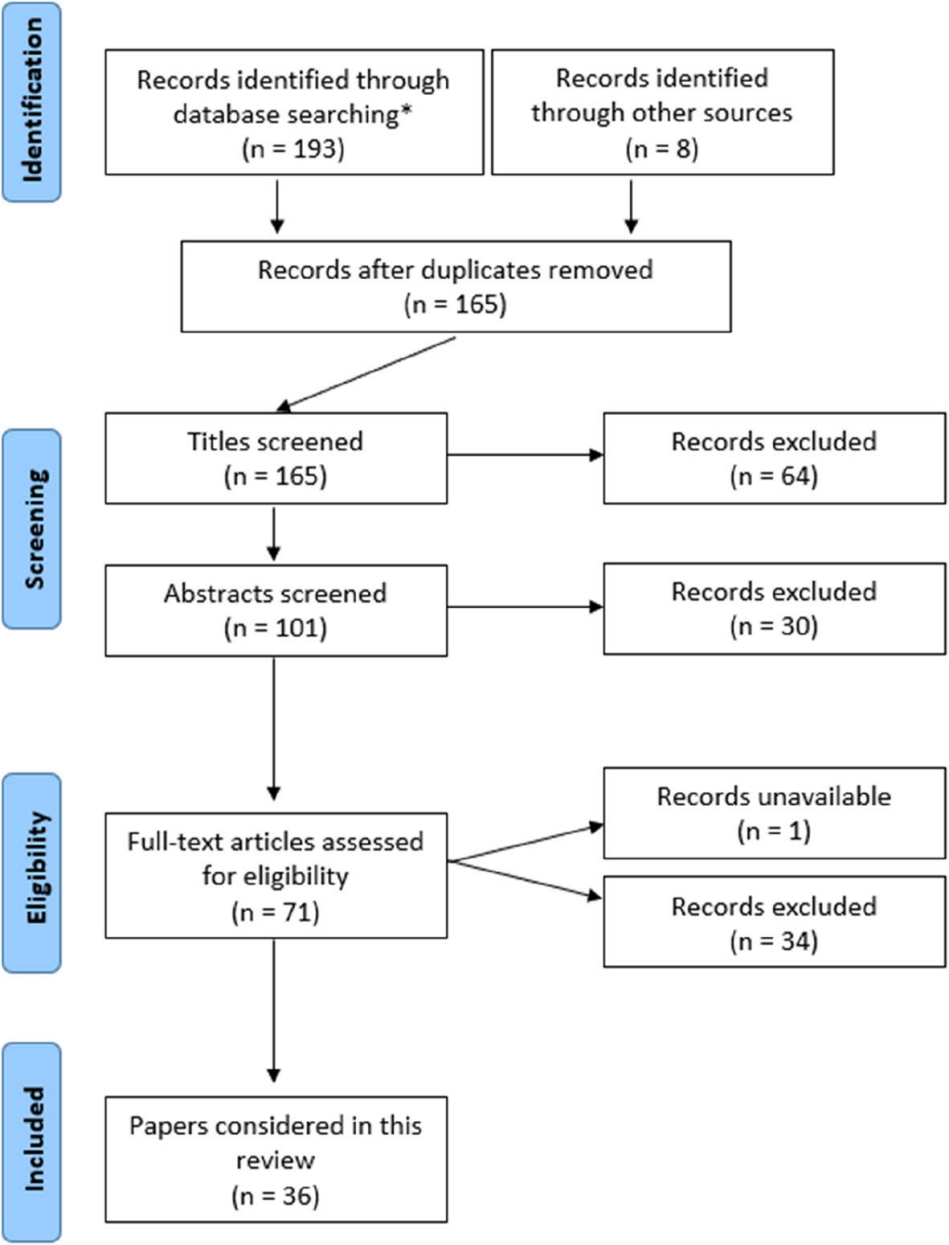
PRISMA search summary * Sat Feb 18 22:41:10 2023 Search: [(cobalt) OR (chromium)] AND [(orthopaedic*) OR (orthopedic*) OR (implant)] AND [(allergy) OR (immuno*) OR (hypersensitivity)] AND (English\[Language])

To ensure a relevant, accurate and representative summarization of the current state of understanding of immune-mediated allergy to implanted cobalt and/or chromium-containing metal implants, a structured and systematic search and retrieval of publications were performed according to the accepted Preferred Reporting Items for Systematic Reviews and Meta-Analyses (PRISMA) guidelines. The search results are depicted in Fig. 1. Three databases: (i) Cochrane; (ii) EMBASE; and (iii) Medline were searched from inception until 18 February 2023. Search results were limited, in the first instance, to articles available in the English language with available abstracts. The following MESH terms were used: [(cobalt) OR (chromium)] AND [(orthopedic*) OR (orthopaedic*) OR (implant)] AND [(allergy) OR (immuno*) OR (hypersensitivity)]. Titles and abstracts of identified records were screened to exclude obviously irrelevant studies. Articles that described in vivo, in vitro or topical applications of cobalt/chromium compounds, and/or specifically immunological testing of such materials were reviewed. No limitations were placed on age, gender, date, type of study, or length of follow-up. Articles were excluded if they did not specifically discuss or report cobalt/chromium-based allergic reactions/reactivity, or if no full-text in English language was available. The bibliographies of relevant papers were manually reviewed to identify further studies, with additional data coming from international joint registries.

Initially, 193 articles were identified during preliminary database searching. After the exclusion of duplicates, articles that did not match the search intent (i.e., papers not specifically exploring content related to cobalt/chromium implant hypersensitivities) and articles not available in full text form, 71 full-text papers were manually reviewed. At the end of the review process, 36 articles were deemed appropriate for inclusion. As a relatively new topic in the field, there existed a lack of quantitative research within the domain, thus preventing formal “meta-analysis”. With the preserved intent of providing an updated summarization of the topic, a structured review of the identified literature was performed by following meta-synthesis principles.

## Pathophysiology of cobalt-chromium hypersensitivity reactions

From an allogenic perspective, despite commonly being deemed a composite material, the metals cobalt and chromium must be considered separately given their individual propensity for immune system activation. Histologically, local tissue specimens collected at the time of component revision surgery [7, 8] suggested that adverse in vivo reactions to either metal are most commonly akin to type IV hypersensitivity reactions [9]. A summary of the original Gell and Coomb’s hypersensitivity classification is shown in Table 1 [10]. By definition, this subclassification infers the need for initial exposure and immune-reactivity resulting in sensitization, before subsequent hypersensitivity reactions. At cellular level, the underlying pathogenesis of such metal reactions can be subclassified further into macrophage-dominant (CD4 +) [6] or lymphocyte-dominant (CD8 +) cell-mediated pathway activation. Immune-driven ALTRs associated with implanted joint replacements appear to follow the latter mechanisms [5, 11, 12]. There now exists an increasingly robust body of international evidence that supports the premise of a genetic predisposition to such reactions at individual patient level [5, 12, 13]. Certain underlying genotypes may potentiate poor down-regulatory capacity of such reactions, which may result in high-grade ALVAL formation. At their extreme, high-grade ALVALs may form highly-destructive frank pseudotumours [5].

**Table 1:** Hypersensitivity reactions

	**Mediator**	**Physiological function**	**Condition examples**
Type I	IgE	Response against parasites via mast cells and basophil activation	Asthma Anaphylaxis
Type II	IgG/IgM	Cytotoxicity—targets antigens expressed on infected cells	Heparin-induced thrombocytopenia
Type III	Immune complex	Accumulation of antigen–antibody complex formation	Systemic lupus erythematosus
Type IV	Cell-mediated	Four subtype activations: macrophages, eosinophils, cytotoxic CD8 + cells, neutrophils	Contact dermatitis Behcet’s disease

Modified from Gell and Combs [10]

The increased exposure of metal ions and/or biological conjugates within serum and local tissues stands as the most widely accepted proposed mechanism for hypersensitivity reactions to in situ implants [7, 14, 15]. While the bearings of metal-on-metal (MoM) hip replacements were first recognized as major generators of such particulate metal debris [16, 17], recent research has highlighted other potential sources, including the modular junctions of hip and knee replacement components [5], and the head-neck trunnions of THAs, even in the setting of metal-on-polyethylene bearings [18]. While some studies have suggested a direct correlation between elevated serum metal concentrations and subsequent systemic lymphocyte activation, this finding has not been universally observed [19] and the association is widely questioned. Advocates of an immune-mediated pathway for ALTR development believe that metal ions released from implant components sensitize T helper cells, which physiologically serve as antigen-presenting cells, and then lead to lymphocyte and/or macrophage activation and aggressive chemoattractant signalling. With direct respect to immunogenicity, current evidence suggests that cobalt may be a more ready foreign trigger than chromium [20, 21].

## Sensitizing effect

Given an accepted requirement for immune system “priming” prior to type IV reaction [22], sensitization to cobalt/chromium likely only develops following a physiological lag after implant insertion surgery and this phenomenon has been demonstrated in multiple clinical studies [14, 23–25]. Frigerio and colleagues (2011) reported a 7% increase of patients with positive patch tests 1 year after index arthroplasty [25]. The later study by Krecisz (2012) demonstrated an increased incidence of 10.4% (5 patients out of 48) 2 years after the operation [21]. In that study, cobalt demonstrated an increase of 6.7% whilst chromium showed an increase of 3.3% [21]. Subjects with in situ implants had a 3-fold increased reactivity to chromium when compared to matched control groups [26]. While certainly thought-provoking, these studies were all limited by small sample cohort sizes. However, their findings did support the suggestion of a “sensitizing” period before evolution of hypersensitivity features. Interestingly and in contrast, in one recent paper by Münch and colleagues (2015), the delayed prevalence of metal allergy was actually shown to be lower in a small cohort of 64 patients who were patch-tested after THA when compared to the preoperative state [27]. This led the authors to postulate that, in some instances, systemic exposure may actually result in progressive “tolerance” rather than amplified hypersensitivity.

## Self-reporting

Previous investigations suggested no difference in outcomes when comparing patients with self-reported metal allergies versus matched controls in various arthroplasty populations. Schmidt et al. (2019), and Kennon et al. (2020), demonstrated this in TKR and shoulder arthroplasties, respectively [28, 29]. The latter, despite generalizability again being limited by sample size, reported sensitivity features collected via patient-completed questionnaire in 32 of 46 of the included patients despite no overt signs of associated complications [30]. While the current role of patient-reported outcome measures (PROMs) is widely accepted and relevant data are often routinely collected, the lack of an established diagnostic gold standard for the diagnosis/confirmation of genuine intrinsic metal allergy has, to date, limited scientifically-robust correlation between the two. Accepting that PROMs likely play an influential role in patients’ willingness to consider/seek revision surgery, such future data may be of value in patient-supported decision-making.

## Serum ions

The level of serum metal ions has not been proven to correlate with increased hypersensitive reaction. Multiple studies have shown that serum cobalt/chromium levels were elevated post implant. However, they were not correlated specifically with metal allergy [17, 31]. To our knowledge, only one study demonstrated a direct causal link between in vivo metal exposure and subsequent lymphocyte reaction [19]. That study reported the contrast between soluble metal ion exposure and measurable lymphocyte reactivity when comparing healthy volunteers, a cohort of patients with advanced osteoarthritis, and two separate groups with in situ metal-on-metal and metal-on-polyethylene THAs. After matching for general demographics, the study demonstrated a difference in intrinsic cell-mediated immune reactivity between groups. The authors reported a near-linear relationship between in vivo metal ion levels and lymphocyte reactivity and suggested a tentative clinical correlation between cellular immune stimulation and ion exposure levels. The findings of this paper are yet to be reproduced in other settings and require further validation. Thus, these investigation techniques may play a role in the diagnosis of suspected hypersensitive reactions to cobalt/chromium implants, but should be utilized in combination with other investigations and clinical assessments.

## Skin patch testing (PT)

Historically, in the absence of a more reliable means, skin-based “patch testing” has been widely employed as a surrogate for determination of patient allergic to metals [32]. Such testing may be neither accurate nor reliable for many metals commonly found in joint replacements given poor transdermal penetration and hence limited direct lymphocyte exposure [31, 33]. In spite of this recognized shortcoming, the rates of positive patch tests in detecting cutaneous hypersensitivity reactions reportedly varied significantly for both metals, ranging from 1%–9% for cobalt and 1%–4% for chromium on a population-wide scale [20, 32]. A positive patch test criterion has been recommended by the International Contact Dermatitis Research Group, involving erythema, infiltration and papules of varying severity [34]. Hypersensitive reactions are different between deep and cutaneous tissues, primarily mediated by lymphocytes or macrophages at deep layers, as opposed to Langerhans cells at superficial layers. Patch testing before surgery correlates poorly with implant outcomes and does not allow for reliable prediction of subsequent hypersensitivity reactions to orthopaedic implants [21, 27, 28, 31, 35–43].

It is important to recognize that a large proportion of patients with positive patch tests to cobalt and/or chromium are often asymptomatic [31]. As suggested above, this likely reflects the pathophysiological differences between cutaneous “irritative” processes and deeper tissue (i.e., periprosthetic) immune activation. One recent, small, prospective study of 87 patients undergoing either THA or TKA demonstrated metal allergy in 47 subjects through in vitro cellular activation testing but did not show a relationship between these results and clinically-meaningful allergy and short-term postoperative pain [37]. Another study examined patients with knee/hip arthroplasties, compared the results of symptomatic and asymptomatic patients, and found that 24 of 102 and 13 of 92, respectively, to be metal allergic [41]. Krecisz et al. (2012), performed a 2-month postoperative evaluation of patients and found 10.4% of participants complained of symptoms in keeping with implant intolerance, despite 25% showing contact allergy (16.7% cobalt, 8.3% chromium) [21]. Desai’s (2019) reviewed TKR patients three months postoperativelly and found that while 16% of their patients were positive for skin patch tests, only 12% of the cohort were symptomatic [4]. This 25% disparity range again raises the concern regarding the reliability of patch testing as a definitive diagnostic means.

In ankle open reduction and internal fixation surgeries, So and colleagues (2011) found no difference between positive and negative patch test reactions (and history of metal hypersensitivity) in terms of American Academy of Orthopaedic Surgeons (AAOS) scores (34 vs. 32, *P* = 0.73) [42]. The main limiting factor was the relatively small sample size, with only 27 included patients patch-tested. These results did, however, appear to be consistent with evidence generated in the setting of shoulder arthroplasties. A retrospective study following up 40 patients over 2 years showed that 40% of patients had allergic symptoms, either formally diagnosed or self-reported. However, formal PT was only positive in 4 patients and, of these none showed clinical or radiographic signs of implant complications [31]. Similarly, Kennon et al. (2020), reported on 13 shoulder arthroplasty patients undergoing PT, with only one patient yielding negative result. Despite the 12 positive reactions (i.e., 92.3%), the patients still reported an improvement in function and pain relief following surgery even if implants contained the culprit metals [28].

Overall, previous studies yielded consistent findings regarding patch tests both preoperatively and postoperatively, suggesting that they are a poor predictor of implant outcomes and should perhaps be utilized with caution in the attempts to diagnose hypersensitivity reactions to implants.

## MELISA/LTT

Lymphocyte proliferation testing (LPT) is a specific immune-associated investigation which measures proliferation of T-cells on stimulation with certain exogenous triggers. Lymphocyte transformation testing (LTT) is a test that measures cell-mediated response to a specific antigen. While the terms LPT and LTT are often used interchangeably in immunological practice given both measure reactive T-cell proliferation, in antigen-induced metal allergy testing, LTT result would become a more accurate indicator.

The LTT is interpreted through a stimulation index (SI), which represents the ratio of incorporated activity in a stimulated sample versus a non-stimulated control culture. An SI cut-off of ≥ 2–3 has been used by the majority of studies as a positive LTT result. However, currently, there is no consensus regarding the ideal cut-off and the absence of agreement is compounded by a lack of true “gold standard” indicator [33]. Further investigation in this area is needed to establish interpretive guidelines to support wider adoption. A modified form of LTT, known as memory lymphocyte immune-stimulation assay (MELISA), is specifically used to determine the proliferation of peripheral blood lymphocytes following incubation with metal ions. On this basis, MELISA is therefore specifically used in an attempt to quantitatively diagnose type 4 hypersensitivity reactions to metal ions and may represent a more sensitive testing option than conventional standards when periprosthetic allergy is being considered.

Previous studies reported the use of LTT/MELISA alongside patch testing to assist in the diagnosis of hypersensitivity reactions to prostheses [35, 44]. Thomas et al. (2015) reported the results of PT, LTT and periprosthetic histology in a cohort of 25 patients with TKAs containing cobalt, chromium and molybdenum but showed poor correlation between tests [45]. The recent results reported by Bracey et al. (2022), also revealed discordance between the testing modalities within the same patients [33]. Under current utilization conditions, there are currently no evidence -based guidelines surrounding LTT (and MELISA).

## Peri-operative screening

A study utilizing the Danish Hip Arthroplasty Registry (DHAR) identified 356 individuals who had patch-test associated dermatitis following primary THA performed for uncomplicated osteoarthritis [38]. Gender-matched controls (712) from the patch test database were sought for each case. The study could only demonstrate that revision prevalence was similar in cases who were operated prior to patch-testing and in patients after patch testing [38]. Another study, using a similar screening technique for patients from the same national registry, compared the prevalence of contact (i.e., skin) allergy to nickel, chromium and cobalt between arthroplasty patients with and without revision surgery. They reported no meaningful between-group differences. However, in a subsequent secondary analysis, they were able to show that the prevalence of cobalt and chromium (but not nickel) allergy was “markedly higher” in patients undergoing a second or subsequent revision [27]. The authors concluded that while metal allergy diagnosed with skin patch testing prior to index surgery did not appear to be directly associated with a subsequent risk of implant failure or revision rates, the pathogenesis was likely to be multifactorial and influenced by other, as yet undefined, considerations.

There are currently no validated nor universally accepted screening methods for immune-mediated metal allergy. Although patch testing is often utilized in the initial investigations for suspected hypersensitivity reaction to cobalt/chromium arthroplasties [46], it has no role in screening patients prior to their surgery and likely grossly over-estimates the likelihood of a hypersensitivity reaction. If screening was to be considered prior to surgery, current limited evidence would suggest that LTT may be far more suitable and reliable than skin patch testing and better reflect deep tissue hypersensitivity reactions [35], even prior to index exposure and sensitization. Again, further evidence is required to best frame the appropriate utilization of this emerging technology, including general indications and interpretation guidelines. Genetic testing of IL1RN VNTR allele 498 bp can be considered especially for patients with a history of generalized atopy with an up to 4-fold increased risk of symptomatic arthroplasty reported [41]. However, the current evidence base surrounding pre-implant testing utilizing this non-specific marker of atopic predisposition remains poor and has not yet been associated with improved clinical outcomes. At this stage, it cannot be specifically recommended.

## Role for targeted HLA screening

The seminal findings from the ortho-genomic study by Kilb and colleagues (2018) demonstrated a strong genetic predisposition to ALVAL development in patients with in situ metal-on-metal hip replacements who were shown to carry a specific HLA allele variant [12]. This highlighted a potential role for targeted genotype screening for high-risk groups both before and possibly after implant insertion [5]. A subsequent large-scale international collaboration by the same author group was also the first to report the prevalence of high-grade ALVALs or pseudotumours around primary TKAs at the time of aseptic revision surgery (> 7%) [5]. A recent third paper in this series (2023) reported the prevalence of specific HLA genotypes in subjects who developed periprosthetic pseudotumor around hip resurfacings, again suggesting a strong predictive association [13]. On the basis of this developing evidence base, we have also begun routinely requesting targeted HLA screening for our suspected metal allergy patients to demonstrate the presence or absence of the published “at risk” genotype.

## General diagnostic approach

As a primary differential diagnosis, periprosthetic infection must be confidently excluded before consideration for immune-mediated allergy can be safely entertained. There are likely many overlapping clinical features between the two diagnoses. Given the higher order concern associated with infection, we strongly recommend that the investigative pathway towards the diagnosis of metal allergy includes screening for (and reasonable exclusion of) infection. This will include a thorough history, targeted physical examination and routine screening blood tests. In keeping with the current Musculoskeletal Infection Society (MSIS) guidelines, we recommend testing white cell count and differential (including both neutrophil and lymphocyte indices), inflammatory markers, including both CRP and ESR, and both eGFR and serum albumin [47]. We also include routine d-dimer testing although the definitive value in the context of potential metal allergy remains at an early stage of understanding.

In the current setting, sterile solid tissue specimen collection at the time of surgery (be it an arthroscopic, limited open, or open procedure) and subsequent analysis by an experienced histopathologist stands as the closest measure to a definitive/gold standard in diagnosing hypersensitivity reactions [45]. Ideally, specimens are retrieved from an anatomically diverse series of locations at the immediate implant-host interfaces without the potentially confounding effect of current or recent antibiotic therapy. This can be a highly skill- and user-dependent procedure that carries with it a not insignificant risk profile (including general anaesthetic and operative risks, iatrogenic introduction of infection, damage to the in situ implant/bond, and false negative results due to poor sampling technique). There is also usually a considerable cost associated with this invasive testing approach. On account of all of these important considerations, surgical sampling may not represent the most appropriate and ethical means of investigating potential allergy for the majority of patients.

**Fig. 2:**
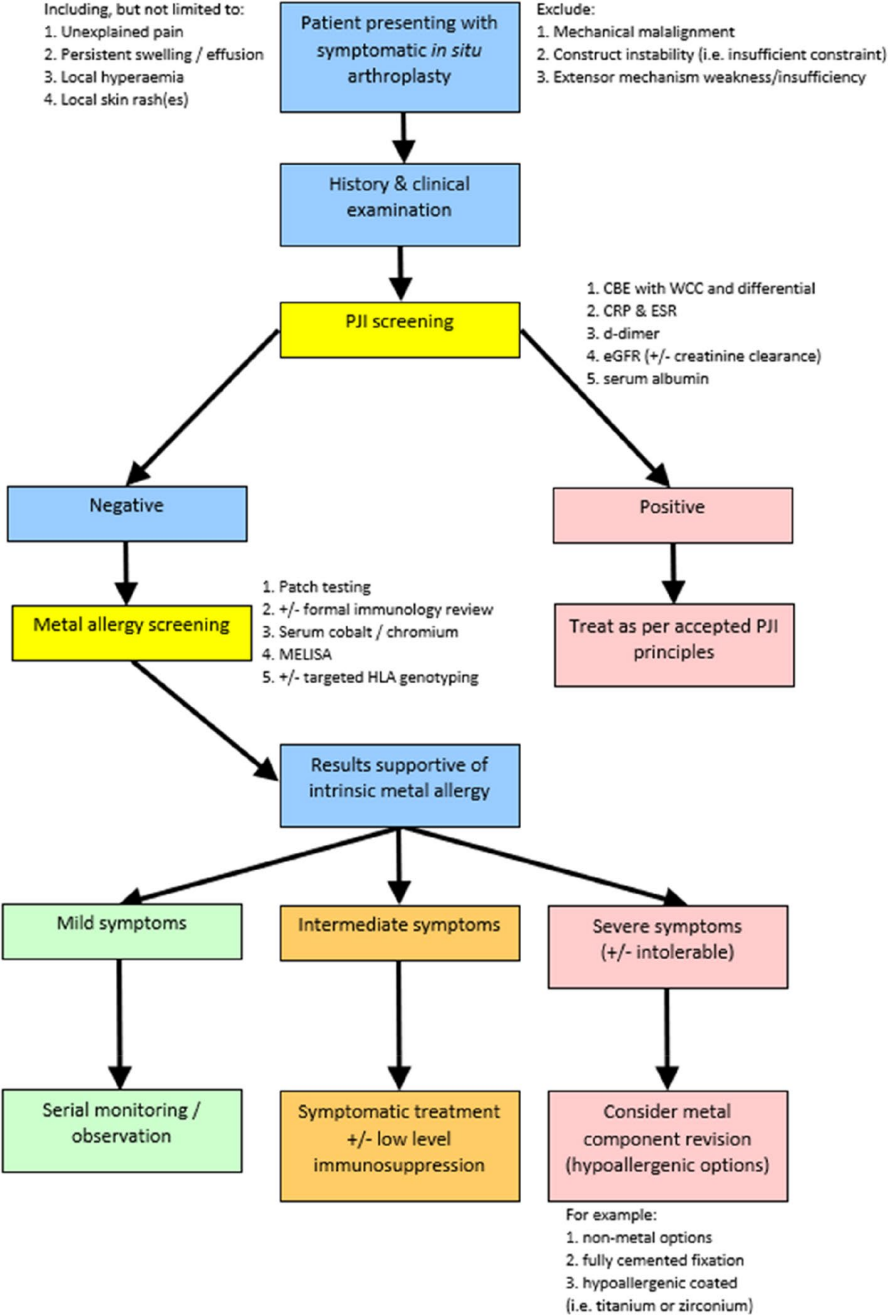
Proposed investigation pathway — clinically-suspected intrinsic metal allergy

Through non-surgical means, if the former screening blood tests and clinical assessment do not support infection in an otherwise immunocompetent host, then a hypersensitivity work-up should be considered. Under current conditions, until the establishment of a sensitive and accepted “gold standard” diagnostic method, metal hypersensitivity may therefore be considered somewhat a diagnosis of exclusion. Therefore, there exists great potential value in the wider adoption of standardized screening approaches to limit the detrimental biasing effect of inter-clinician variability (undoubtedly otherwise influenced by personal training, experience and local resource access). Despite their objective lack of sensitivity and specificity, the results of previously performed patch testing may still be considered in the overall context of the patient’s clinical picture, although we see no value in otherwise ordering this specific testing method prospectively. Where available, we have been performing serum cobalt and chromium assays for all patients being considered for allergy to either metal [44, 45] and have been screening for the non-specific HLA marker, B27. Elevated serum ion levels have shown inconsistent association with hypersensitivity reactions [26] when used as a primary diagnostic determinant but may hold weighted value in composite assessment. Given the lack of international guidelines for testing for hypersensitivity reactions to cobalt/chromium and the current lack of an accepted non-surgical “gold standard” testing approach, we have devised a local guideline (Fig. 2) to assist this diagnostic dilemma. Our guideline considers pre-investigative clinical impressions (pre-test probability), targeted history and physical examination, and screening serum blood testing. Where available, we do see value in adding MELISA testing although we acknowledge that this relatively new technology may not be widely available and may be prohibitively expensive in other settings for use as a screening tool.

## Management

Given a current lack of absolute binary diagnostic testing for immune-mediated hypersensitization to cobalt and/or chromium, a symptomatic patient with sufficient suspicion on clinical grounds, including interpretation of the before-described screening results, should be informed of the material-related risks for revision surgery and the potential need to use of a hypoallergenic implant. In particular, such counselling should include the possibility of a postoperative lack of symptomatic improvement. A recent study (2022) by Bracey and colleagues reported minimal overall improvements after revision TKA using “hypoallergenic” implants in patients with suspected metal hypersensitivity [33]. Reporting the findings of a small cohort of 28 primary and 20 separate revision TKA patients, the authors found poor correlation between available pre-surgery metal allergy screening approaches (including “skin patch” and lymphocyte testing methods) and subsequent clinician- and patient-defined outcomes. Again, the lack of an accepted (or proposed) gold standard screening method undermines the presented findings with a lack of objective confirmation of the true presence of intrinsic allergy. While making a valuable contribution to the limited knowledge base in this area, the authors did not report correlative histopathological findings of intraoperatively collected surgical solid tissue specimens, which may have otherwise been of opportunistic value. Ultimately, the decision to entertain consideration for major revision surgery (versus less invasive or non-operative measures) must be weighed up on the basis of acknowledgement of the intrinsic risks and potential benefits.

In the specific subgroup in which patients are being considered for index primary joint replacement and metal allergy to cobalt or chromium is clinically-supported, current evidence suggests that a hypoallergenic implant coating may reduce immunogenic metal ion release and thus reduce the potential risk of sensitization and immune priming [22]. Hypoallergenic coatings have been reported as “safe” in the short-, medium- and long-term by previous authors [48–50]. Separate studies by Lutzner et al. [48] and Postler et al. [50] reported similar outcomes from formal RCTs measuring 5- and 10-year patient-reported outcomes correlated with serum metal ion levels when contrasting/comparing coated (i.e., hypoallergenic) and standard TKAs. Of note, only cobalt levels showed a slight increase versus the control group of Lutzner’s investigation, but these elevations were interpreted as having not reached clinically-relevant threshold [48].

## Review limitations

We acknowledge that this work has several important limitations. Firstly, we recognize that, as a fledgling domain within the field of arthroplasty, the published evidence base is limited in both scope and levels of evidence. Indeed, much of the work we have reviewed represented small case series and largely uncontrolled comparative investigations. Scientifically robust high-tier (i.e., level 1 and level 2) evidence is simply not yet available. As with all retrospective reviews of the literature, the “quality” of our work is inherently linked to the quality of the index papers. Due to a lack of standardization in metal allergy diagnostic methods, we were unable to perform formal meta-analyses. At best, our work may therefore be considered a meta-synthesis of the current literature. Nonetheless, by working out an up-to-date and thorough summarization of the modern literature, we hope readers will find value in what we present and conclude. Additionally, given the inconsistent standard of studies available for review, we have not been able to formally and objectively consider the potential for inherent bias in the key results. Finally, as with many other developing areas of arthroplasty surgery, the generalizability and validity of findings remain to be established by using the methods in a diverse range of clinical settings. We hope that our structured approach to the assessment and evaluation of the patients with a suspected intrinsic metal allergy may be of some value to future endeavors by standardizing investigative approaches and reporting conventions.

## Future research

Our review has highlighted several promising areas of potential future research related to immune-mediated metal allergy testing. Firstly, framing the optimized utility of in vitro cellular activation testing (such as LTT/MELISA) appears an important step. The information garnered from such analysis should confirm both the diagnostic accuracy and recommendations for standardized result interpretation. One could envisage that the preliminary diagnostic value could be compared to an invasive surgical specimen collection sampling “gold” standard although it would also be tempting to add a third diagnostic arm to provide definitive evidence to either flatly refute a role for skin patch testing or clarify this common held conception. Secondarily, utilitarianistic cost-efficacy data must be generated to allow for the establishment of expense versus benefit conclusions. This will help to define the breadth of screening use which will likely be of value in determining target population groups. Thereafter, the application of such testing can be explored in two distinct patient populations: symptomatic patients with an in situ joint replacement (presumed aseptic) and those being considered for an index replacement, in whom reasonable clinical grounds exist to suspect an immune-mediated allergy to cobalt or chromium. Once a new diagnostic convention has been established, consideration can be given to optimized hypoallergenic implant (and revision implant) selection, including alloy metallurgy and barrier coating consideration. The associated increased costs with the use of such specialized implants must also be factored into decision-making paradigms. Finally, the expanding evidence basis for targeted HLA genetic screening/genotyping appears to be gaining international momentum, how best to incorporate the results of such testing into more standardized diagnostic and management pathways also requires further due consideration.

## Conclusion

Cobalt and chromium are common metals found in conventional orthopaedic implants and are typically well tolerated. It is increasingly recognized, however, that a not insignificant subset of patients exposed to these metals develop some degree of clinically-meaningful allergic reaction. Such reactions may manifest as pain, local swelling and/or effusion, implant-host bond loosening or the local development of a spectrum of inflammatory tissue reactions (e.g., ALTRs). No accepted, mainstream, diagnostic test for genuine immune reaction to in situ metal medical devices currently exists. The historical standard of skin “patch testing” has been shown to be a grossly unreliable diagnostic means and positive results likely reflect cutaneous direct “irritation” with little (if any) parallels with periarticular (i.e., deep tissue) reactions. While surgical specimen collection from implant-host interfaces and subsequent high-quality histopathological assessment is/are likely the true “most accurate” diagnostic standard, it is a highly-invasive process that is heavily user (i.e., surgeon)-dependent and carries considerable potential risk. From a non-operative perspective, based on the best available current evidence, currently, a combination of targeted (routine) screening blood tests with the additional metal (antigen) and specific in vitro analyses using MELISA or LTT appears to be the most appropriate regime. Where available, targeted patient-specific HLA genotyping may add further diagnostic confidence. These screening blood results can be considered in the context of a pre-test probability based on clinical suspicion supported by appropriate patient history and physical examination findings. The cost and availability of such testing, however, is likely to be regionally variable and may alone preclude wider early uptake. Many exciting avenues for future research exist within this novel field, the findings of which will undoubtedly shape optimized utilization and result interpretation.

## Data Availability

Not applicable.

## Abbreviations

ALVAL: Aseptic lymphocyte-dominated vasculitis-associated lesions
ALTR: Adverse local tissue reaction
ARMD: Adverse reaction to metal debris
LTT: Lymphocyte transformation testing
LPT: Lymphocyte proliferation testing
MELISA: Memory lymphocyte immune-stimulation assay
TKA: Total knee arthroplasty
THA: Total hip arthroplasty
HLA: Human leukocyte antigens
